# Tumor infiltrating lymphocytes and change in tumor load on MRI to assess response and prognosis after neoadjuvant chemotherapy in breast cancer

**DOI:** 10.1007/s10549-024-07484-7

**Published:** 2024-09-16

**Authors:** L. M. Janssen, B. B. L. Penning de Vries, M. H. A. Janse, E. van der Wall, S. G. Elias, R. Salgado, P. J. van Diest, K. G. A. Gilhuijs

**Affiliations:** 1 Image Sciences Institute, University Medical Centre Utrecht, Utrecht University, Heidelberglaan 100, 3584 CX Utrecht, The Netherlands; 2 Julius Center for Health Sciences and Primary Care, University Medical Centre Utrecht, Utrecht University, Utrecht, The Netherlands; 3 Department of Medical Oncology, University Medical Centre Utrecht, Utrecht University, Utrecht, The Netherlands; 4 Department of Pathology, ZAS Hospitals, Antwerp, Belgium; 5 Division of Research, Peter Mac Callum Cancer Centre, Melbourne, Australia; 6 Department of Pathology, University Medical Centre Utrecht, Utrecht University, Utrecht, The Netherlands

**Keywords:** Breast cancer, Tumor infiltrating tumor cells, Magnetic resonance imaging, Neoadjuvant chemotherapy, Pathological complete response

## Abstract

**Purpose:**

In this study, we aimed to explore if the combination of tumor infiltrating lymphocytes (TILs) and change in tumor load on dynamic contrast-enhanced magnetic resonance imaging leads to better assessment of response to neoadjuvant chemotherapy (NAC) in patients with breast cancer, compared to either alone.

**Methods:**

In 190 NAC treated patients, MRI scans were performed before and at the end of treatment. The percentage of stromal TILs (%TILs) was assessed in pre-NAC biopsies according to established criteria. Prediction models were developed with linear regression by least absolute shrinkage and selection operator and cross validation (CV), with residual cancer burden as the dependent variable. Discrimination for pathological complete response (pCR) was evaluated using area under the receiver operating characteristic curves (AUC). We used Cox regression analysis for exploring the association between %TILs and recurrence-free survival (RFS).

**Results:**

Fifty-one patients reached pCR. In all patients, the %TILs model and change in MRI tumor load model had an estimated CV AUC of 0.69 (95% confidence interval (CI) 0.53–0.78) and 0.69 (95% CI 0.61–0.79), respectively, whereas a model combining the variables resulted in an estimated CV AUC of 0.75 (95% CI 0.66–0.83). In the group with tumors that were ER positive and HER2 negative (ER+/HER2−) and in the group with tumors that were either triple negative or HER2 positive (TN&HER2+) separately, the combined model reached an estimated CV AUC of 0.72 (95% CI 0.60–0.88) and 0.70(95% CI 0.59–0.82), respectively. A significant association was observed between pre-treatment %TILS and RFS (hazard ratio (HR) 0.72 (95% CI 0.53–0.98), for every standard deviation increase in %TILS, *p* = 0.038).

**Conclusion:**

The combination of TILs and MRI is informative of response to NAC in patients with both ER+/HER2− and TN&HER2+ tumors.

**Supplementary Information:**

The online version contains supplementary material available at 10.1007/s10549-024-07484-7.

## Introduction

The neoadjuvant treatment approach for breast cancer involves systemic therapy followed by a post-neoadjuvant phase consisting of surgery and/or radiotherapy and/or systemic therapy such as endocrine treatment. Neoadjuvant chemotherapy (NAC) with the tumor in situ allows tumor-response monitoring in vivo using, for instance, dynamic contrast-enhanced magnetic resonance imaging (DCE-MRI). After surgery, the actual response of the tumor is established from the excised tissue, as expressed by pathological complete response (pCR) or the residual cancer burden (RCB), which is associated with survival [1–3]. Unfortunately NAC often comes with side effects, some of them long-lasting [4, 5]. Breast surgery has its own side-effects such as unsatisfactory cosmetic outcome, which is in turn correlated with reduced quality of life [6, 7]. Reducing these side effects could be possible in patients with tumors sensitive to chemotherapy, where a good response might also be achieved with less intensive NAC, and surgery could perhaps be omitted or postponed. In order to safely select patients for (participation in clinical trials on) de-escalation of NAC or surgery, methods for accurate prediction of response to NAC are essential. Although many efforts have been made to develop new methods, both invasive and non-invasive, none have yet been considered adequate to incorporate in clinical practice.

In the post-neoadjuvant phase following surgery (and in most cases radiotherapy), the question on who is to benefit from additional systemic treatment also cannot be answered with full confidence yet. Efforts have been made to develop methods to predict patient survival based on the tumor and patient characteristics such as the Nottingham Prognostic index or PREDICT, and gene expression tests like Oncotype DX, MammaPrint, EndoPredict and Breast Cancer Index [8, 9]. The majority of these methods has, however, been based on patients who received breast surgery as initial treatment and their role following NAC has not been fully established yet [2, 3]. There is thus still a clinical need for further refinement of post-neoadjuvant treatment decisions.

Many known predictors for response to NAC and post-neoadjuvant prognosis relate to tumor characteristics like estrogen receptor (ER) and human epidermal growth factor receptor 2 (HER2) status [2], tumor grade [10]) and TNM stage [11]. In addition, the immunogenic microenvironment of the tumor plays a role in sensitivity to treatment [12]. TILs are usually less abundant in ER+/HER2− breast cancer, and conflicting results have been reported about the relationship with response to NAC and prognosis after NAC [13]. Because the percentage of TILs is easily scored on hematoxylin and eosin (HE) slides of a diagnostic tumor biopsy, additional research to evaluate its predictive value in the ER+/HER2− groups is warranted.

To improve prediction of response, an appealing approach is to combine information from different available sources. One study has suggested the potential of TILs added to pre-treatment radiomics of DCE-MRI to better predict response to NAC [14]. Although promising, this was a relatively small study in only TN breast cancer patients. Hence, the potential of this combination needs further confirmation in TN breast cancer, whereas its value is yet to be explored in the other breast cancer subtypes.

Hence the aim of this study is to explore if the combination of TILs and DCE-MRI improves the assessment of response to NAC and if TILs enable stratification of prognosis in the post-neoadjuvant phase in the whole group and two subgroups of patients: those with tumors that were ER positive and HER2 negative (ER+/HER2−) and those with tumors that were either triple negative or HER2 positive (TN&HER2+).

## Methods

### Patients

Two patient cohorts were combined in this study. Cohort A consisted of patients with stage 1–3 invasive breast cancer of any subtype treated with NAC followed by surgery at the University Medical Center Utrecht between January 1st 2011 and December 1st 2019. Patients with oligometastatic disease treated with curative intent were also included. Patients were excluded if they received less than two cycles of NAC, or if biopsies nor MR images were available. All patients with an indication for adjuvant endocrine therapy were offered this treatment.

Cohort B consisted of patients with invasive breast cancer from a prospective multicenter NAC study, running from 2020 to 2022. All patients signed informed consent before enrollment. Inclusion criteria were: female patients aged 18 years or older, histologically proven invasive breast carcinoma and planned to receive NAC. Exclusion criteria were grade 1 estrogen receptor (ER)-positive and HER2-negative breast cancer, inflammatory breast cancer, distant metastases on positron emission tomography/computed tomography (PET/CT), prior ipsilateral breast cancer < 5 years ago, other active malignant diseases in the past 5 years (excluding squamous cell or basal cell carcinoma of the skin), pregnancy or lactation and contra-indications for MRI. All patients underwent NAC according to Dutch guidelines [15] and were offered adjuvant endocrine therapy if indicated. The potential of MRI features of cohort B to assess response to NAC in combination with liquid biopsies has been reported previously [16].

### Pathological evaluation

The percentage of stromal TILs (%TILs) in the pre-treatment biopsies and surgical resection specimen were assessed by two experienced breast pathologists (RS, PvD), following the International TILs Working Group guideline [17]. The pathologists were blinded to the outcome and non-pathologic predictors during %TILs assessment. The residual cancer burden (RCB) [1] was assessed by an experienced breast pathologist (PvD) using the calculator provided by the MD Anderson website in both cohorts [18]. The pathologist was blinded to the %TILs and non-pathology predictors during RCB assessment. For cohort A, the tumor grade (according to the Nottingham modification of the Bloom and Richardson method [6, 7]) was extracted from the pathology report. If the grade was not available from the report, the biopsy was reassessed for grade (by PvD). Pre-treatment ER, HER2 and nodal status were extracted from the pathology reports as well. For cohort B, central revision of all biopsies was performed (by PvD). Positive nodal status was established by image guided lymph node fine needle aspiration or biopsy, or sentinel node procedure prior to NAC. PCR was defined as ypT0/isN0.

### MR imaging

MRI scans were performed before start of treatment in both cohorts. For the patients from cohort A, the MRI that was performed closest to surgery was used as the end-of-treatment MRI. For the patients from cohort B, the end-of-treatment MRI scan was performed after NAC was completed.

For both the pre-treatment and end-of-treatment scans, the following five features were calculated: (1) number of lesions, defined as the number of lesions that were delineated (2) total lesion volume, in mm^3^ (3) mean lesion volume, defined as total lesion volume divided by the number of lesions (4) total largest diameter, defined as the largest diameter spanning all lesions (5) sum of the largest diameters, defined as the sum of the largest diameters of individual lesions. In the data pre-processing step, a variable was created for the relative difference in each of the five MRI tumor load features, by dividing the end-of-treatment feature value by the pre-treatment feature value (hereafter: change in MRI tumor load). Lesions were segmented as follows: In cohort A, semi-automated delineation of the breast lesions was performed according to previously reported method based on histopathology-validated region growing [19]. In short, seed points were placed by an experienced biomedical engineer (MHAJ) in the lesions based on clinical reporting and verified by a radiologist. From this seed point, automated constrained volume growing took place based on contrast uptake. Small manual corrections were made to remove erroneous segmentations of vessels. In cohort B, a previously validated deep learning-based approach was employed based on the nnU-Net framework [20]. Only segmentations in the breast with the biopsy proven tumor were taken into account.

The DCE-MRI protocol consisted of five post-contrast series, taken at a 60 to 90 s interval in cohort A and a minimum of three post-contrast acquisitions in cohort B. If less than five post-contrast series were available, the last available post-contrast series was used as substitute for the missing series. Only the T1-weighted dynamic contrast series were analyzed in this study. In cohort A, imaging was performed using either 1.5 T or 3 T MRI scanners from a single vendor (Philips), while in cohort B, imaging was exclusively performed on 3 T scanners from multiple vendors. Fat-suppression was applied to all scans.

### Statistical analysis

The MRI variables and %TILs values were transformed into variables with normal-shaped distributions using a Box-Cox procedure. To be able to use cases with incomplete data in the model development and evaluation, we imputed missing values. For cohort A and B, if the end-of-treatment tumor load features were missing and they were available at an earlier point (from a scan during treatment), the earlier time-point feature values were used for imputation by last observation carried forward. If the features were only available at one timepoint, the sample mean was used for the change in tumor load features. The %TILs and RCB were imputed with the sample mean.

Different prediction models were developed to explore the possible additional value of predictors: a model with only %TILs, a model with only change in MRI tumor load, and a third model combining change in MRI tumor load and %TILs. The individual %TILs and change in MRI tumor load models were developed and evaluated in the whole patient population, and then evaluated in the ER+/HER2− and triple negative (TN)/HER2+ subgroups separately. Each model was a (main effects) linear regression model with RCB as the dependent variable, fit using L1-penalised maximum likelihood estimation (LASSO) with the penalty parameter set at the value that yielded the lowest mean squared error in an inner-loop tenfold cross validation scheme. To estimate the expected out-of-sample performance of the various models in terms of discrimination, we used an additional outer-loop cross validation (CV) with fivefolds and 10 repeats. Discrimination for pCR (RCB 0) vs. residual disease (RCB > 0) was evaluated using receiver operating characteristic (ROC) curves and, in particular, the area under the ROC curve (AUC). 95% confidence intervals (CI) were estimated by bootstrapping the original data and performing repeated cross-validation in each bootstrap sample.

For estimation of the median follow-up time after NAC, the reverse Kaplan Meier method was used. For exploring the association between %TILs and recurrence-free survival (RFS, as previously defined [21]), we used Cox regression analysis with the box-cox transformed %TILs as the explaining variable, calculating hazard ratios (HR). In order to create Kaplan Meier curves, the original %TILs values were stratified in two predefined groups of 1–10% and > 10% TILs, corresponding to the low vs. intermediate + high groups of a large pooled analysis [22]. We merged the intermediate and high subgroups of TILs because of the expected lower TILs counts in the ER+/HER2− group. All statistical analysis were performed in R software version 4.2.2.

## Results

A total of 190 patients were included in this study (129 in cohort A, 61 in B) of which 106 were ER+/HER2−, 40 ER−/HER2 (TN)− and 44 ER+−/HER2+. All patients underwent surgery after NAC, 51 patients reached pCR. Median follow up time after NAC was 58 months. There were a total of 31 RFS events (Table 1).

**Table 1:** Patient and tumor characteristics of breast cancer patients treated with NAC that were included in the study, overall and according %TILs and cohort

		Total (*N* = 190)	%TILs 1–10* (*N* = 122)	%TILs > 10* (*N* = 68)	Cohort A (*N* = 129)	Cohort B (*N* = 61)
Age (years)	Median [Min, Max]	50 [25–78]	52.50 [25.00, 78.00]	48.50 [25.00, 71.00]	50.00 [25.00, 78.00]	50.00 [25.00, 72.00]
Histology (%)	Invasive carcinoma NST	132 (69.5)	79 (64.8)	53 (77.9)	79 (61.2)	53 (86.9)
	Ductolobular carcinoma	33 (17.4)	26 (21.3)	7 (10.3)	31 (24.0)	2 (3.3)
	Lobular carcinoma	20 (10.5)	15 (12.3)	5 (7.4)	15 (11.6)	5 (8.2)
	Other	5 (2.6)	2 (1.6)	3 (4.4)	4 (3.1)	1 (1.6)
Grade (%)	1	17 (8.9)	14 (11.5)	3 (4.4)	17 (13.2)	0 (0.0)
	2	92 (48.4)	64 (52.5)	28 (41.2)	68 (52.7)	24 (39.3)
	3	81 (42.6)	44 (36.1)	37 (54.4)	44 (34.1)	37 (60.7)
	Missing	2	0	2	2	0
IHC subtype (%)	ER−/HER2− (TN)	40 (21.1)	22 (18.0)	18 (26.5)	19 (14.7)	21 (34.4)
	ER+-/HER2+	44 (23.2)	25 (20.5)	19 (27.9)	27 (20.9)	17 (27.9)
	ER+/HER2−	106 (55.8)	75 (61.5)	31 (45.6)	83 (64.3)	23 (37.7)
cT stage (%)	T1	30 (15.9)	19 (15.7)	11 (16.2)	19 (14.7)	11 (18.3)
	T2	111 (58.7)	75 (62.0)	36 (52.9)	74 (57.4)	37 (61.7)
	T3	37 (19.6)	21 (17.4)	16 (23.5)	25 (19.4)	12 (20.0)
	T4 a-b	11 (5.8)	6 (5.0)	5 (7.4)	11 (8.5)	0 (0.0)
	Missing	1	1	0	0	1
Nodal metastases (%)	Absent	90 (47.4)	60 (49.2)	30 (44.1)	67 (51.9)	23 (37.7)
	Present	100 (52.6)	62 (50.8)	38 (55.9)	62 (48.1)	38 (62.3)
%TILs*	1–10	122 (64.2)			76 (58.9)	46 (75.4)
	> 10	68 (35.8)			53 (41.1)	15 (24.6)
Neoadjuvant treatment (%)	FEC	2 (1.1)	2 (1.6)	0 (0.0)	2 (1.6)	0 (0.0)
	FEC-DOC	79 (41.6)	53 (43.4)	26 (38.2)	79 (61.2)	0 (0.0)
	DOC + CP	3 (1.6)	1 (0.8)	2 (2.9)	3 (2.3)	0 (0.0)
	TAC	1 (0.5)	0 (0.0)	1 (1.5)	1 (0.8)	0 (0.0)
	AC-P	42 (22.1)	28 (23.0)	14 (20.6)	16 (12.4)	26 (42.6)
	AC-P + trastuzumab	24 (12.6)	11 (9.0)	13 (19.1)	24 (18.6)	0 (0.0)
	AC-P + carboplatin	18 (9.5)	12 (9.8)	6 (8.8)	0 (0.0)	18 (29.5)
	Paclitaxel + trastuzumab	2 (1.1)	2 (1.6)	0 (0.0)	1 (0.8)	1 (1.6)
	PTCP	18 (9.5)	12 (9.8)	6 (8.8)	2 (1.6)	16 (26.2)
	AC + 2 × intensified CP, thiotepa and carboplatin	1 (0.5)	1 (0.8)	0 (0.0)	1 (0.8)	0 (0.0)
Relative change on MRI* median [min, max]	Number of lesions	63.7 [0.0, 400.0]	66. 7 [0.0, 400.0]	50.0 [0.0, 250.0]	66.7 [0.0, 200.0]	50.0 [0.0, 400.0]
	Total volume	4.5 [0.0, 221.0]	6.7 [0.0, 115.6]	1.6 [0.0, 221.0]	6.0 [0.0, 221.0]	3.8 [0.0, 63.2]
	Mean volume	6.5 [0.0, 221.0]	9.0 [0.0, 97.2]	1.6 [0.0, 221.0]	6.5 [0.0, 221.0]	6.6 [0.0, 71.4]
	Total largest diameter	44.2 [0.0, 425.3]	50.6 [0.0, 425.3]	23.5 [0.0, 289.4]	426 [0.0, 425.3]	47.1 [0.0, 328.1]
	Sum largest diameter	34.9 [0.0, 192.9]	41.6 [0.0, 192.9]	17.9 [0.0, 135.8]	41.3 [0.0, 192.9]	31.9 [0.0, 135.8]
Surgery type (%)	Lumpectomy	99 (52.1)	66 (54.1)	33 (48.5)	74 (57.4)	25 (41.0)
	Mastectomy	91 (47.9)	56 (45.9)	35 (51.5)	55 (42.6)	36 (59.0)
RCB*	Median [min, max]	1.54 [0.00, 4.22]	1.61 [0.00, 3.72]	1.23 [0.00, 4.22]	1.55 [0.00, 4.22]	1.22 [0.00, 3.42]

cT stage: clinical tumor stage according to the American Joint Committee on Cancer (AJCC) staging system

*FEC* 5-fluorouracil, epirubicin, cyclophosphamide, *DOC* docetaxel, *CP* cyclophosphamide, *AC-P* Doxorubicin, cyclophosphamide and paclitaxel, *PTCP* Paclitaxel, trastuzumab, carboplatin and pertuzumab, *TAC* Docetaxel, doxorubicin and cyclophosphamide, *AC* Doxorubicin, cyclophosphamide

*Values after imputation

Pearson’s correlation coefficient of %TILs in biopsy with response variables were as follows: − 0.21, − 0.08, − 0.01, − 0.06, − 0.1 and − 0.12 for RCB, the relative change in number of lesions, total volume, mean volume, total largest diameter and sum largest diameter, respectively. Illustration of MRI images and descriptives of two typical patients can be found in Supplementary Fig. 1.

There were a total of 13% missing values for the change in tumor load MRI variables (15% in cohort A and 10% in cohort B) and 15% for the %TILs values (22% in cohort A and 0% in cohort B). RCB was missing in 2 cases. See Supplementary Table 2 and 3 for missing data per cohort. Proportion of missing data was comparable among the IHC subtypes.

### Assessment of response to NAC

**Fig. 1:**
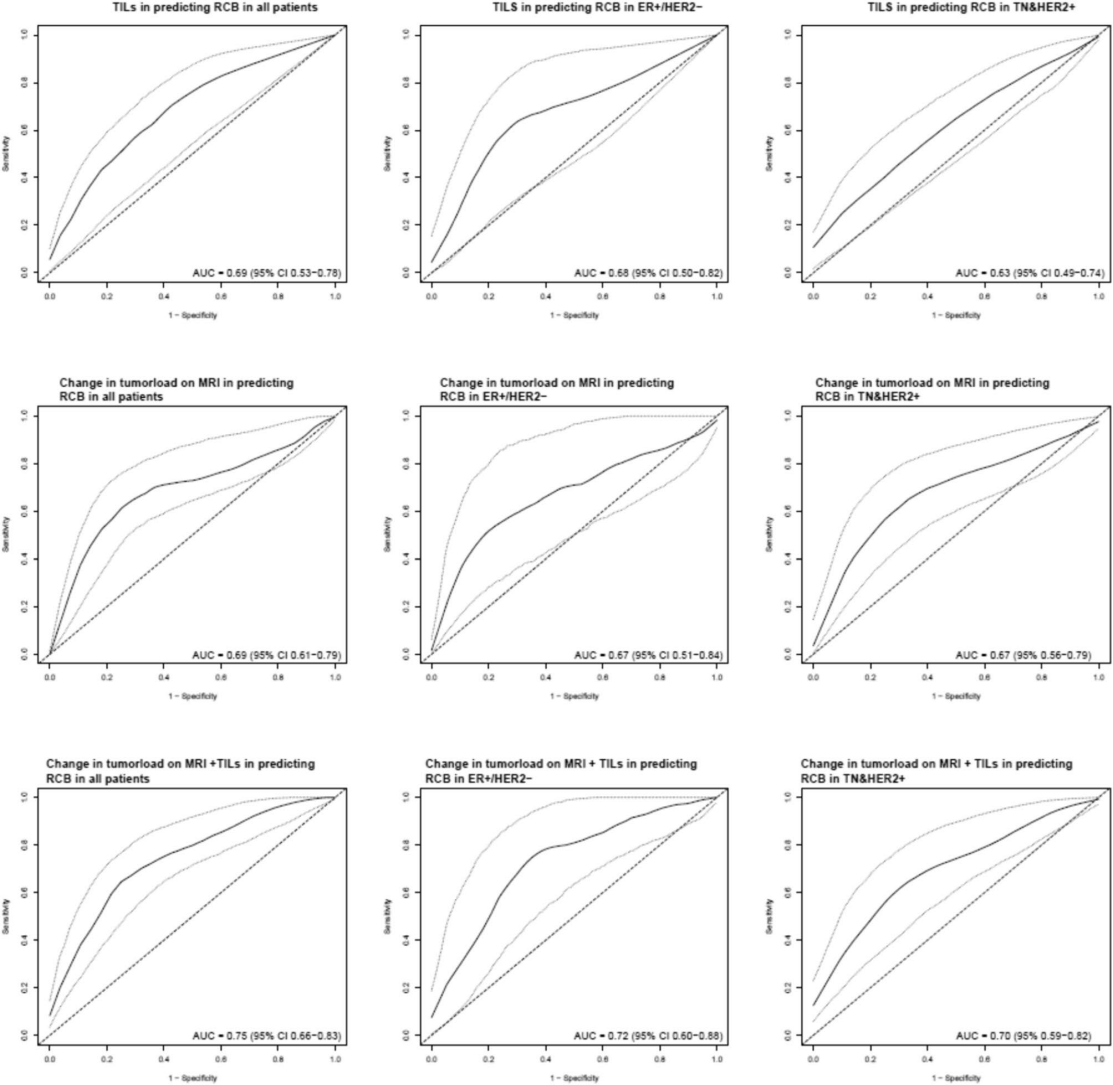
ROC curves for discriminating between pCR (RCB 0) and residual disease (RCB > 0) for the different prediction models. The black line represents the mean curve over all CV loops. The dotted lines represent the 95% confidence intervals. *AUC* cross-validated area under the curve. *CI* confidence interval

Figure 1 depicts the ROC curves and corresponding (cross validated) AUC of each of the models. Coefficients can be found in Supplementary Table 4. A prediction model containing the change in MRI tumor load reached an estimated CV AUC of 0.69 (95% CI 0.61–0.79) in all patients, and a model with only %TILs had an estimated CV AUC of 0.69 (95% CI 0.53–0.78). A prediction model combining %TILs and change in MRI tumor load had an estimated CV AUC of 0.75(95% CI 0.67–0.83). The change in MRI tumor load model evaluated in the ER+/HER2− patient group yielded an estimated CV AUC of 0.67(95% CI 0.51–0.84), the %TILs-only model an estimated CV AUC of 0.68 (95% CI 0.50–0.82), while the combined model had an estimated CV AUC of 0.72 (95% CI 0.60–0.88). For the TN&HER2+ subgroup, the change in MRI tumor load model had an estimated CV AUC of 0.67 (95% CI 0.56–0.79), the %TILs only model an estimated CV AUC of 0.63 (95% CI 0.49–0.74), while the combined model had an estimated CV AUC of 0.70(95% CI 0.59–0.82).

### Explorative analysis of TILs vs. event-free survival

**Fig. 2:**
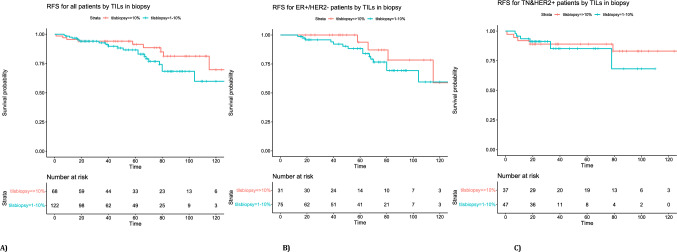
Kaplan Meier curve for recurrence free survival stratified to TILs in biopsy of > 10% (red line) vs. 1–10% (blue line). **A** RFS for all patients by TILs in biopsy. **B** RFS for ER+/HER2− patients by %TILs. **C** RFS for TN&HER2+ patients by %TILs

%TILs was significantly associated with RFS in all patients (HR 0.72 (95% CI 0.53–0.98), for every standard deviation increase in %TILS, *p* = 0.038). This association does not appear substantially different in the two subgroups (HR 0.68 (95% CI 0.44–1.074), *p* = 0.10 in the ER+/HER2− group and HR 0.72 (95% CI 0.44- 1.19), *p* = 0.20 in the TN&HER2+ group). Figure 2 shows the survival curves of patients with %TILs 1–10 vs. > 10 in each of the groups. %TILs in the resection specimen was also evaluated but was not available in all patients resulting in insufficient data to properly assess the association with RFS.

## Discussion

In this multicenter study, we explored the combination of %TILs and change in tumor load on DCE-MRI to assess response to NAC in patients with ER+/HER2− and TN&HER2+ breast cancer. A higher CV AUC was observed for the combination of %TILs and change in tumor load on MRI compared to either one alone in the whole group. This was also observed in the ER+/HER2− group, as well as in the TN&HER2+ group.

The difference in observed discriminative ability should, however, be interpreted with caution given the wide confidence intervals.

There is a large need for improvement of response prediction, before clinical trials on postponing or omitting surgery after NAC have a good chance of succeeding [23]. Our work suggests that %TILs and MRI may hold complementary information and could be a useful combined biomarker for response to NAC in different breast cancer subtypes.

TILs have been shown by others to be correlated to pCR in the HER2+ and TN subtypes, with higher TILs relating to higher pCR rates [12, 22]. In the ER+/HER2− subtype, the literature is inconclusive. A large pooled analysis by Denkert et al. showed a significant positive correlation between TILs and pCR in the ER+/HER2− subtype[22]. A different meta-analysis and some other smaller studies did, however, not find this correlation [12, 13, 24–26]. TILs are reported to be less frequent in ER+/HER2− breast cancer compared to the other subtypes [27], which makes it less likely to find a correlation in smaller groups. We found an association between TILs and response to NAC as measured by RCB in the whole group of patients. One study found significant correlations between RCB classes and TIL CD8/FOXP3 ratio in TN breast cancer [28]. A different study by Elmahs et al. did not find a correlation between TILs and RCB class, perhaps due to small sample size [29].

In both the TN subtype and, more recently, in the luminal subtype as well, the combination of NAC with immunotherapy was shown to improve pCR [30–32]. In both groups treated with this combination, higher sTILs were associated with higher pCR rates [33–36]. Personalizing treatment and not giving more than necessary is of special importance in the light of high costs associated with immunotherapy. Models like the one presented in this study could potentially aid clinical decision making in treatment with the combination of NAC and immunotherapy in the future. They should however be evaluated in a population treated with this regimen.

With regard to the prognostic value of TILs, we found that higher %TILs in biopsy is associated with better RFS after NAC in the whole cohort. This suggests that %TILs could also be useful for post NAC decision making, although its role in relation to other prognostic factors was not investigated here due to too few events. High TILs have been reported to be correlated to better prognosis in the TN and HER2+ subtypes [12, 22, 37]. In the ER+/HER2− group, the pooled analysis by Denkert et al. reported low TILs (0–10%) to be correlated with improved disease free survival, in contrast with our results [22]. The meta-analysis by Li et al. reported no correlation between TILs and survival [12]. Our work thus contributes to the growing body of research on the prognostic role of TILs in breast cancer. We did not have enough data to evaluate the relationship with TILs in the residual tumor to RFS, but work is underway to incorporate TILs in the residual tumor after NAC in the RCB to further stratify post-neoadjuvant prognosis [38]. Since MRI is more accurate in evaluating response to NAC compared to mammography, ultrasound and physical examination, it is widely used in clinical practice [39–41]. Radiological assessment alone is, however, not accurate enough to guide treatment decisions [42]. A (semi-) automated method for evaluating response to NAC could be of interest, since manual measurement by RECIST is associated with intra- and interobserver variability [43–45].

Tissue biopsy is always a part of the diagnostic pre-treatment work-up and assessing TILs in the biopsy is quick and easily implemented, possibly even more so when artificial intelligence algorithms are deployed [46]. The combination of TILs and computer extracted MRI features may therefore be an efficient use of information that is available from the clinical workflow without additional (invasive) procedures. Our results suggest the complementary value of these different data sources in assessing response to NAC, which could ultimately help in sparing patients unnecessary treatment.

Our study has several limitations. First, there was no independent cohort to perform external validation of the developed models. Second, due to limited sample size, we were unable to account for relevant predictors such as treatment regimen and nodal status, or to evaluate the HER2+ and TN subtypes separately. A larger sample size and longer follow up could result in more events and thus a more robust EFS analysis. Third, our two cohorts contain patients from different periods in time, which resulted in different treatment regimens that may not reflect current clinical practice. Additionally, for cohort A, not all biopsies were centrally revised for ER, HER2 and nodal status. This could result in unwanted interobserver variability in these variables, which is however a reflection of clinical practice. Lastly, the MRI processing differed between cohort A and B. In theory, this could have impacted the results, although the methods have been shown to lead to highly correlated results [20].

In conclusion, our results show that the combination of TILs and change in tumor load on MRI is informative of response after NAC overall, as well as in the ER+/HER2− and TN&HER2+ groups separately. This could be of interest for clinical trials on de-escalating surgical intervention. More work is, however, needed to reduce uncertainty and improve accuracy by modifying for other predictors as well.

## Supplementary Information

Below is the link to the electronic supplementary material.

Supplementary file1 (DOCX 241 KB)

## Data Availability

Datasets and R code used for analysis are available from the corresponding author on reasonable request.

## References

[1] Symmans WF, Peintinger F, Hatzis C, Rajan R, Kuerer H, Valero V et al (2007) Measurement of residual breast cancer burden to predict survival after neoadjuvant chemotherapy. J Clin Oncol 25(28):4414–4422 17785706 10.1200/JCO.2007.10.6823

[2] Cortazar P, Zhang L, Untch M, Mehta K, Costantino JP, Wolmark N et al (2014) Pathological complete response and long-term clinical benefit in breast cancer: the CTNeoBC pooled analysis. Lancet 384(9938):164–172 24529560 10.1016/S0140-6736(13)62422-8

[3] Yau C, Osdoit M, van der Noordaa M, Shad S, Wei J, de Croze D et al (2021) Residual cancer burden after neoadjuvant chemotherapy and long-term survival outcomes in breast cancer: a multicentre pooled analysis of 5161 patients. Lancet Oncol 23:149 34902335 10.1016/S1470-2045(21)00589-1 PMC9455620

[4] Hassett MJ, O’Malley AJ, Pakes JR, Newhouse JP, Earle CC (2006) Frequency and cost of chemotherapy-related serious adverse effects in a population sample of women with breast cancer. JNCI: J Natl Cancer Inst 98(16):1108–1117 16912263 10.1093/jnci/djj305

[5] Azim HA, de Azambuja E, Colozza M, Bines J, Piccart MJ (2011) Long-term toxic effects of adjuvant chemotherapy in breast cancer. Ann Oncol 22(9):1939–1947 21289366 10.1093/annonc/mdq683

[6] Hennigs A, Biehl H, Rauch G, Golatta M, Tabatabai P, Domschke C et al (2016) Change of patient-reported aesthetic outcome over time and identification of factors characterizing poor aesthetic outcome after breast-conserving therapy: long-term results of a prospective cohort study. Ann Surg Oncol 23(5):1744–1751 26545376 10.1245/s10434-015-4943-z

[7] Dahlbäck C, Ullmark JH, Rehn M, Ringberg A, Manjer J (2017) Aesthetic result after breast-conserving therapy is associated with quality of life several years after treatment. Swedish women evaluated with BCCT.core and BREAST-Q™. Breast Cancer Res Treat 164(3):679–687 28536951 10.1007/s10549-017-4306-5 PMC5495840

[8] Andre F, Ismaila N, Allison KH, Barlow WE, Collyar DE, Damodaran S et al (2022) Biomarkers for adjuvant endocrine and chemotherapy in early-stage breast cancer: ASCO guideline update. J Clin Oncol 40(16):1816–1837 35439025 10.1200/JCO.22.00069

[9] Candido Dos Reis FJ, Wishart GC, Dicks EM, Greenberg D, Rashbass J, Schmidt MK et al (2017) An updated PREDICT breast cancer prognostication and treatment benefit prediction model with independent validation. Breast cancer Res: BCR 19(1):58 28532503 10.1186/s13058-017-0852-3 PMC5440946

[10] Lips EH, Mulder L, de Ronde JJ, Mandjes IA, Koolen BB, Wessels LF et al (2013) Breast cancer subtyping by immunohistochemistry and histological grade outperforms breast cancer intrinsic subtypes in predicting neoadjuvant chemotherapy response. Breast Cancer Res Treat 140(1):63–71 23828499 10.1007/s10549-013-2620-0 PMC3706735

[11] Giuliano AE, Connolly JL, Edge SB, Mittendorf EA, Rugo HS, Solin LJ, et al. (2017) Breast Cancer-Major changes in the American Joint Committee on Cancer eighth edition cancer staging manual. CA Cancer J Clin 67(4):290–303 10.3322/caac.21393 28294295

[12] Li S, Zhang Y, Zhang P, Xue S, Chen Y, Sun L, Yang R (2022) Predictive and prognostic values of tumor infiltrating lymphocytes in breast cancers treated with neoadjuvant chemotherapy: a meta-analysis. Breast 66:97–109 36219945 10.1016/j.breast.2022.10.001 PMC9550538

[13] Goldberg J, Pastorello RG, Vallius T, Davis J, Cui YX, Agudo J et al (2021) The immunology of hormone receptor positive breast cancer. Front Immunol. 10.3389/fimmu.2021.674192 34135901 10.3389/fimmu.2021.674192 PMC8202289

[14] Jimenez JE, Abdelhafez A, Mittendorf EA, Elshafeey N, Yung JP, Litton JK et al (2022) A model combining pretreatment MRI radiomic features and tumor-infiltrating lymphocytes to predict response to neoadjuvant systemic therapy in triple-negative breast cancer. Eur J Radiol 149:110220 35193025 10.1016/j.ejrad.2022.110220

[15] (IKNL) IkN. Landelijke richtlijn Borstkanker 2020 [Available from: https://richtlijnendatabase.nl

[16] Janssen LM, Janse MHA, Penning de Vries BBL, van der Velden BHM, Wolters-van der Ben EJM, van den Bosch SM et al (2024) Predicting response to neoadjuvant chemotherapy with liquid biopsies and multiparametric MRI in patients with breast cancer. NPJ Breast Cancer 10(1):10 38245552 10.1038/s41523-024-00611-z PMC10799888

[17] Salgado R, Denkert C, Demaria S, Sirtaine N, Klauschen F, Pruneri G et al (2015) The evaluation of tumor-infiltrating lymphocytes (TILs) in breast cancer: recommendations by an International TILs working group 2014. Ann Oncol 26(2):259–271 25214542 10.1093/annonc/mdu450 PMC6267863

[18] Center MAC. Residual Cancer Burden Calculator [cited 2018. Available from: http://www3.mdanderson.org/app/medcalc/index.cfm?pagename=jsconvert3

[19] Alderliesten T, Schlief A, Peterse J, Loo C, Teertstra H, Muller S, Gilhuijs K (2007) Validation of semiautomatic measurement of the extent of breast tumors using contrast-enhanced magnetic resonance imaging. Invest Radiol 42(1):42–49 17213748 10.1097/01.rli.0000248849.99014.7e

[20] Janse MHA, Janssen LM, van der Velden BHM, Moman MR, Wolters-van der Ben EJM, Kock MCJM et al (2023) Deep learning-based segmentation of locally advanced breast cancer on MRI in relation to residual cancer burden: a multi-institutional cohort study. J Magn Reson Imaging 58(6):1739–1749 36928988 10.1002/jmri.28679

[21] Hudis CA, Barlow WE, Costantino JP, Gray RJ, Pritchard KI, Chapman JA et al (2007) Proposal for standardized definitions for efficacy end points in adjuvant breast cancer trials: the STEEP system. J Clin Oncol 25(15):2127–2132 17513820 10.1200/JCO.2006.10.3523

[22] Denkert C, von Minckwitz G, Darb-Esfahani S, Lederer B, Heppner BI, Weber KE et al (2018) Tumour-infiltrating lymphocytes and prognosis in different subtypes of breast cancer: a pooled analysis of 3771 patients treated with neoadjuvant therapy. Lancet Oncol 19(1):40–50 29233559 10.1016/S1470-2045(17)30904-X

[23] van Hemert AKE, van Duijnhoven FH, van Loevezijn AA, Loo CE, Wiersma T, Groen EJ, Peeters M (2023) Biopsy-guided pathological response assessment in breast cancer is insufficient: additional pathology findings of the MICRA trial. Ann Surg Oncol 30:4682 37071235 10.1245/s10434-023-13476-6 PMC10319687

[24] Ono M, Tsuda H, Shimizu C, Yamamoto S, Shibata T, Yamamoto H et al (2012) Tumor-infiltrating lymphocytes are correlated with response to neoadjuvant chemotherapy in triple-negative breast cancer. Breast Cancer Res Treat 132(3):793–805 21562709 10.1007/s10549-011-1554-7

[25] Hwang HW, Jung H, Hyeon J, Park YH, Ahn JS, Im YH et al (2019) A nomogram to predict pathologic complete response (pCR) and the value of tumor-infiltrating lymphocytes (TILs) for prediction of response to neoadjuvant chemotherapy (NAC) in breast cancer patients. Breast Cancer Res Treat 173(2):255–266 30324273 10.1007/s10549-018-4981-x

[26] Russo L, Maltese A, Betancourt L, Romero G, Cialoni D, De la Fuente L et al (2019) Locally advanced breast cancer: tumor-infiltrating lymphocytes as a predictive factor of response to neoadjuvant chemotherapy. Eur J Surg Oncol 45(6):963–968 30745134 10.1016/j.ejso.2019.01.222

[27] Loi S, Sirtaine N, Piette F, Salgado R, Viale G, Van Eenoo F et al (2013) Prognostic and predictive value of tumor-infiltrating lymphocytes in a phase III randomized adjuvant breast cancer trial in node-positive breast cancer comparing the addition of docetaxel to doxorubicin with doxorubicin-based chemotherapy: BIG 02–98. J Clin Oncol 31(7):860–867 23341518 10.1200/JCO.2011.41.0902

[28] Miyashita M, Sasano H, Tamaki K, Chan M, Hirakawa H, Suzuki A et al (2014) Tumor-infiltrating CD8+ and FOXP3+ lymphocytes in triple-negative breast cancer: its correlation with pathological complete response to neoadjuvant chemotherapy. Breast Cancer Res Treat 148(3):525–534 25395319 10.1007/s10549-014-3197-y

[29] Elmahs A, Mohamed G, Salem M, Omar D, Helal AM, Soliman N (2022) The impact of tumor infiltrating lymphocytes densities and Ki67 index on residual breast cancer burden following neoadjuvant chemotherapy. Int J Breast Cancer 2022:2597889 36133828 10.1155/2022/2597889 PMC9484975

[30] Schmid P, Cortes J, Pusztai L, McArthur H, Kümmel S, Bergh J et al (2020) Pembrolizumab for early triple-negative breast cancer. N Engl J Med 382(9):810–821 32101663 10.1056/NEJMoa1910549

[31] Loi S, Curigliano G, Salgado RF, Romero Diaz RI, Delaloge S, Rojas C et al (2023) LBA20 A randomized, double-blind trial of nivolumab (NIVO) vs placebo (PBO) with neoadjuvant chemotherapy (NACT) followed by adjuvant endocrine therapy (ET) ± NIVO in patients (pts) with high-risk, ER+ HER2− primary breast cancer (BC). Ann Oncol 34:S1259–S1260

[32] Cardoso F, McArthur HL, Schmid P, Cortés J, Harbeck N, Telli ML et al (2023) LBA21 KEYNOTE-756: Phase III study of neoadjuvant pembrolizumab (pembro) or placebo (pbo) + chemotherapy (chemo), followed by adjuvant pembro or pbo + endocrine therapy (ET) for early-stage high-risk ER+/HER2− breast cancer. Ann Oncol 34:S1260–S1261

[33] Wood SJ, Gao Y, Lee JH, Chen J, Wang Q, Meisel JL, Li X (2024) High tumor infiltrating lymphocytes are significantly associated with pathological complete response in triple negative breast cancer treated with neoadjuvant KEYNOTE-522 chemoimmunotherapy. Breast Cancer Res Treat 205:193 38286889 10.1007/s10549-023-07233-2

[34] Loibl S, Untch M, Burchardi N, Huober J, Sinn BV, Blohmer JU et al (2019) A randomised phase II study investigating durvalumab in addition to an anthracycline taxane-based neoadjuvant therapy in early triple-negative breast cancer: clinical results and biomarker analysis of GeparNuevo study. Ann Oncol 30(8):1279–1288 31095287 10.1093/annonc/mdz158

[35] Schmid P, Salgado R, Park YH, Muñoz-Couselo E, Kim SB, Sohn J et al (2020) Pembrolizumab plus chemotherapy as neoadjuvant treatment of high-risk, early-stage triple-negative breast cancer: results from the phase 1b open-label, multicohort KEYNOTE-173 study. Ann Oncol 31(5):569–581 32278621 10.1016/j.annonc.2020.01.072

[36] Sherene Loi GC, Roberto Salgado, Roberto Iván Romero Díaz,, Suzette Delaloge CIRG, Marleen Kok, Cristina Saura,, Nadia Harbeck EAM, Denise A. Yardley, Lajos Pusztai, Alberto Suárez Zaizar AU, Felipe Ades, Rajalakshmi Chandra, Raheel Nathani MP, Thomas Spires, 16 Jenny Qun Wu, Heather McArthur. Biomarker results in high-risk estrogen receptor‑positive, human epidermal growth factor receptor 2‑negative primary breast cancer following neoadjuvant chemotherapy ± nivolumab: an exploratory analysis of CheckMate 7FL. San Antonio Breast Cancer Symposium; Dec 5–9 20232023

[37] Martín M, Yoder R, Salgado R, Del Monte-Millán M, Alvarez EL, Echavarría I et al (2024) Tumor-infiltrating lymphocytes refine outcomes in triple-negative breast cancer treated with anthracycline-free neoadjuvant chemotherapy. Clin Cancer Res 30:2160 38466643 10.1158/1078-0432.CCR-24-0106 PMC11096004

[38] Dieci MV, Radosevic-Robin N, Fineberg S, van den Eynden G, Ternes N, Penault-Llorca F et al (2018) Update on tumor-infiltrating lymphocytes (TILs) in breast cancer, including recommendations to assess TILs in residual disease after neoadjuvant therapy and in carcinoma in situ: a report of the international immuno-oncology biomarker working group on breast cancer. Semin Cancer Biol 52:16–25 29024776 10.1016/j.semcancer.2017.10.003

[39] Scheel JR, Kim E, Partridge SC, Lehman CD, Rosen MA, Bernreuter WK et al (2018) MRI, clinical examination, and mammography for preoperative assessment of residual disease and pathologic complete response after neoadjuvant chemotherapy for breast cancer: ACRIN 6657 trial. AJR Am J Roentgenol 210(6):1376–1385 29708782 10.2214/AJR.17.18323 PMC6615034

[40] Marinovich ML, Macaskill P, Irwig L, Sardanelli F, Mamounas E, von Minckwitz G et al (2015) Agreement between MRI and pathologic breast tumor size after neoadjuvant chemotherapy, and comparison with alternative tests: individual patient data meta-analysis. BMC Cancer 15:662 26449630 10.1186/s12885-015-1664-4 PMC4599727

[41] Park J, Chae EY, Cha JH, Shin HJ, Choi WJ, Choi YW, Kim HH (2018) Comparison of mammography, digital breast tomosynthesis, automated breast ultrasound, magnetic resonance imaging in evaluation of residual tumor after neoadjuvant chemotherapy. Eur J Radiol 108:261–268 30396666 10.1016/j.ejrad.2018.09.032

[42] Janssen LM, den Dekker BM, Gilhuijs KGA, van Diest PJ, van der Wall E, Elias SG (2022) MRI to assess response after neoadjuvant chemotherapy in breast cancer subtypes: a systematic review and meta-analysis. NPJ Breast Cancer 8(1):107 36123365 10.1038/s41523-022-00475-1 PMC9485124

[43] Beresford MJ, Padhani AR, Taylor NJ, Ah-See ML, Stirling JJ, Makris A et al (2006) Inter- and intraobserver variability in the evaluation of dynamic breast cancer MRI. J Magn Reson Imaging 24(6):1316–1325 17058203 10.1002/jmri.20768

[44] Suzuki C, Torkzad MR, Jacobsson H, Åström G, Sundin A, Hatschek T et al (2010) Interobserver and intraobserver variability in the response evaluation of cancer therapy according to RECIST and WHO-criteria. Acta Oncol 49(4):509–514 20397778 10.3109/02841861003705794

[45] Karmakar A, Kumtakar A, Sehgal H, Kumar S, Kalyanpur A (2019) Interobserver variation in response evaluation criteria in solid tumors 1.1. Acad Radiol 26(4):489–501 29934024 10.1016/j.acra.2018.05.017

[46] Albusayli R, Graham JD, Pathmanathan N, Shaban M, Raza SEA, Minhas F et al (2023) Artificial intelligence-based digital scores of stromal tumour-infiltrating lymphocytes and tumour-associated stroma predict disease-specific survival in triple-negative breast cancer. J Pathol 260(1):32–42 36705810 10.1002/path.6061

