# Supplementary material for: Tumor infiltrating lymphocytes and change in tumor load on MRI to assess response and prognosis after neoadjuvant chemotherapy in breast cancer
Source: Breast Cancer Res Treat. 2024 Sep 16;209(1):167–75. doi: 10.1007/s10549-024-07484-7 (PMC11785616; doi:10.1007/s10549-024-07484-7)
Supplement: Supplementary file 1 — Supplementary file1 (DOCX 241 KB) [file 10549_2024_7484_MOESM1_ESM.docx]

**Supplements**

| Parameter | Cohort A | Cohort B |
| --- | --- | --- |
| Field Strength | 1.5 T, 3 T | 3 T |
| MRI scanner model | Philips Achieva, Ingenia | Philips Ingenia; Siemens MAGNETOM Avanto, Spectra, Skyra or Vida |
| Contrast agent | Gadovist (Bayer) | Dotarem (Guerbet); Gadovist (Bayer); Prohance (Bracco) |
| Voxel volume [mm³] | 0.75 × 0.75 × 0.90  - 0.97 × 0.97 × 1.30 | 0.5 × 0.5 × 0.9 - 1.0 × 1.0 × 1.25 |
| Interval between contrast series [s] | 60 – 90 | 55 – 89 |
| Fat suppression | Yes | Yes |
| Flip angle [°] | 8 - 10 | 8 - 12 |
| Echo time (TE) [ms] | 1.2 - 3.4 | 1.5 - 2.5 |
| Repetition time (TR) [ms] | 3.3 - 7.1 | 3.7 - 5.5 |
| Acquisition plane | Axial | Axial |

***Supplementary Table 1****: MRI characteristics per cohort*

| **Cohort A** | | Missing N (% of total) |
| --- | --- | --- |
| **%TILs** | | 29 (22%) |
| **Relative change on MRI** | Number of lesions | 19 (15%) |
|  | Total volume | 19 (15%) |
|  | Mean volume | 19 (15%) |
|  | Total largest diameter | 19 (15%) |
|  | Sum largest diameter | 19 (15%) |
| **RCB** | | 2 (1.5%) |

***Supplementary Table 2****: missing data in cohort A*

| **Cohort B** | | Missing N (% of total) |
| --- | --- | --- |
| **%TILs** | | 0 (0%) |
| **Relative change on MRI** | Number of lesions | 5 (8%) |
|  | Total volume | 6 (10%) |
|  | Mean volume | 6 (10%) |
|  | Total largest diameter | 6 (10%) |
|  | Sum largest diameter | 6 (10%) |
| **RCB** | | 0 (0%) |

***Supplementary Table 3****: missing data in cohort B*

| Model |  |  |
| --- | --- | --- |
| %TILs | Intercept | 1.8146744  -0.2261445 |
| Change in tumorload on MRI | Intercept  Coefficient relative change in total volume  Coefficient relative change in sum largest diameter  Coefficient relative change in delta number of lesions  Coefficient relative change in total largest diameter  Coefficient relative change in mean volume | 1.4957368  -0.2042496  -0.2411525  0.4914132  .  0.6406588 |
| %TILs + change in tumorload on MRI | Intercept  Coefficient TILs  Coefficient relative change in total volume  Coefficient relative change in sum largest diameter  Coefficient relative change in delta number of lesions  Coefficient relative change in total largest diameter  Coefficient relative change in mean volume | 1.7344652  -0.1692717  .  0.2877455  0.4440148  .  0.4534353 |

***Supplementary Table 4****: intercept and coefficients of the prediction models for all patients*


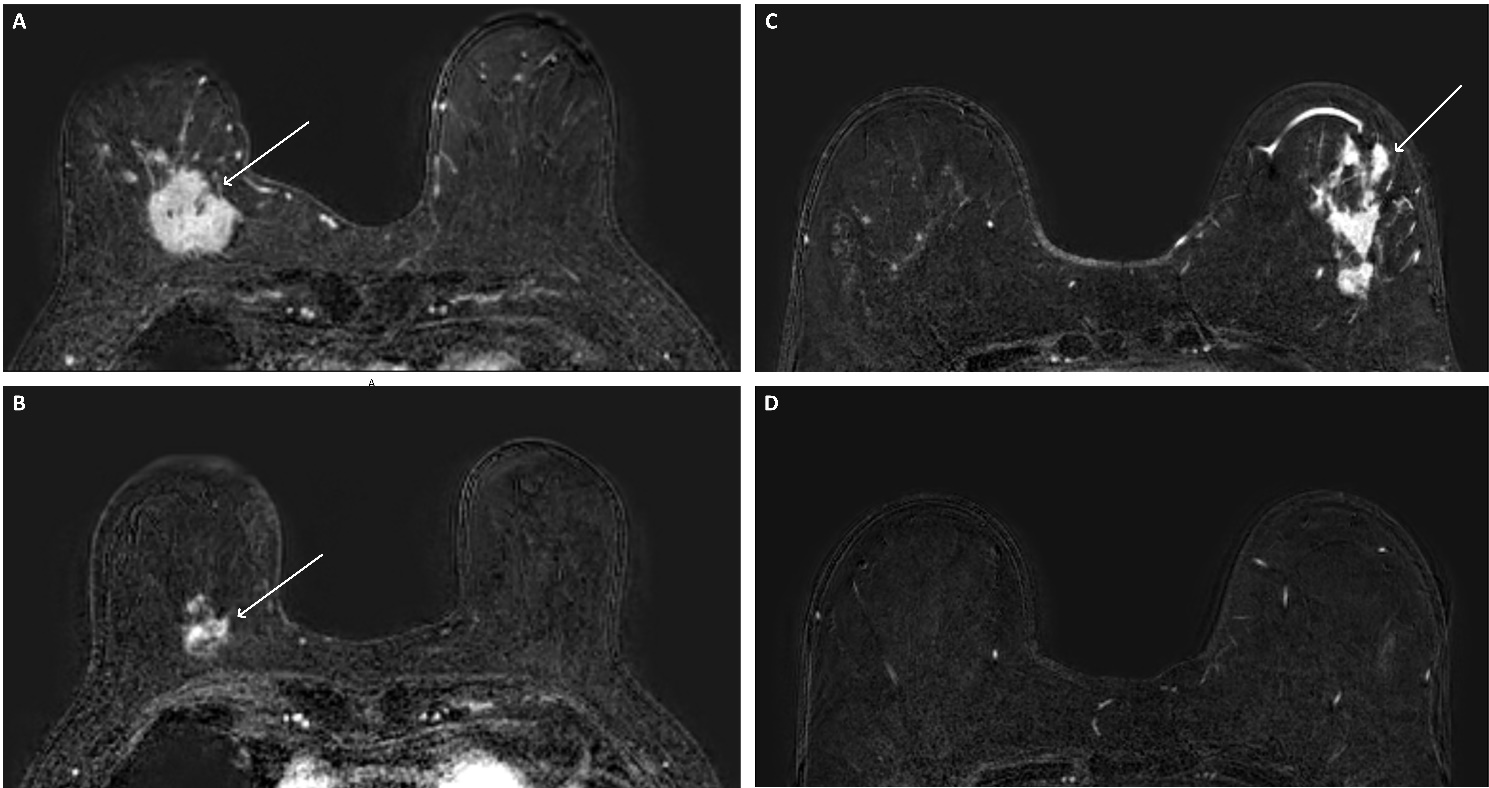


***Supplementary Figure 1****: Illustration of pre-treatment and post-treatment T1-weighted magnetic resonance images of two typical patients. (A) Pre-treatment scan of patient X, tumor infiltrating lymphocytes (TILs) in biopsy are 5%. (B) End of treatment scan of patient X, which shows a partial radiological response, with reduction in total tumor volume to 6.4% of the volume visible on the pretreatment scan. Patient had residual tumor in the resection specimen, residual cancer burden of 2.885. (C) Pre-treatment scan of patient Y. TILs in biopsy are 20%. (D) Post-treatment scan of patient Y, which shows radiological complete response, with a total tumor volume of 0 relative to the pretreatment scan. Patient had pCR in the resection specimen.*
